# Population structure and genetic diversity of *Phakopsora pachyrhizi* in the Southeastern United States

**DOI:** 10.1093/g3journal/jkaf267

**Published:** 2025-12-08

**Authors:** June Clary, Paul M Severns, James W Buck, Robert C Kemerait, Shavannor M Smith

**Affiliations:** Department of Plant Pathology, University of Georgia, Athens, GA 30602, United States; Department of Plant Pathology, University of Georgia, Athens, GA 30602, United States; Department of Plant Pathology, University of Georgia, Griffin, GA 30223, United States; Department of Plant Pathology, University of Georgia, Tifton, GA 31793, United States; Department of Plant Pathology, University of Georgia, Athens, GA 30602, United States

**Keywords:** *Glycine max*, pathogen dispersal, pathogen genetic diversity, *Phakopsora pachyrhizi*, population structure

## Abstract

*Phakopsora pachyrhizi*, the causal agent of soybean rust disease (SBR) on *Glycine max* (soybean), is considered one of the most globally devastating diseases of soybeans and is a particular problem in Brazil, China, Sub-Saharan Africa, and the southern United States. To better understand genetic diversity and epidemiological history of SBR in the United States, 49 *P. pachyrhizi* isolates collected from soybean fields in four Southeastern states (Alabama, Florida, Georgia, and Louisiana) from the 2008 to 2017 growing seasons were genotyped through restriction site-associated genotype by sequencing (GBS). Rarefaction analysis identified 54 informative SNPs among the *P. pachyrhizi* isolates. We found no evidence suggesting sexual or parasexual recombination, and measurements of genetic diversity were low to moderately low. Multiple different statistical approaches, including neighbor-joining trees, K-means hierarchical clustering, discriminant analysis of principal components, and principal coordinates analysis (PCoA) all identified two groups of *P. pachyrhizi* genotypes that associated with geographic location. One group was composed of isolates from south Georgia, and the other with isolates from Alabama, Florida, Georgia (excluding south Georgia), and Louisiana. Our results suggest that two genetically related but distinct genotypes were introduced to the continental United States in a two-phase introduction and overwinter in South Georgia and Florida. The first introduction of one genotype likely occurred in South Georgia in 2004 followed by a later introduction of a second genotype. One genotype remained in South Georgia while the other genotype became established through the Southeastern United States. Future studies are necessary to determine whether SBR in Brazil, China, or Sub-Saharan Africa shows similar patterns of genotype distribution and history or if the United States situation is unique.

## Teaser

Plant pathologists and plant breeders are the target audience for this research. This article provides insight into the genetic diversity and epidemiological history of *Phakopsora pachyrhizi* in North America that is necessary to effectively manage soybean rust. Forty-nine *P. pachyrhizi* isolates collected from soybean fields in four Southeastern states for nine growing seasons were genotyped through genotype by sequencing. Clary *et al*. demonstrated that *P. pachyrhizi* genetic diversity was low to moderately low, it spread to the Northeastern United States by one of two proposed methods, and the Southeastern United States can harbor new genotypes and generate new pathogenic races which may disperse from Florida and Georgia.

## Introduction

*Phakopsora pachyrhizi* is an agronomically important biotrophic fungal pathogen that causes soybean rust (SBR) disease (Goellner et al. 2010). SBR threatens global soybean production and occurs on every soybean-producing continent in the world, with the continental United States having the most recent introduction in 2004 (Schneider et al. 2005). In 2004, SBR was also found on the invasive legume Kudzu (*Pueraria lobata*) in Florida where *P. pachyrhizi* is thought to overwinter (Harmon et al. 2005). The United States Department of Agriculture reported that a total of 398.2 million metric tons of soybeans were produced globally in 2023, with the United States producing 113.34 million metric tons (USDA 2023). *P. pachyrhizi* epidemics have caused annual crop losses of more than $10 billion USD since the first epidemic in Brazil in 2001 (Yorinori et al. 2005; da Silva 2014). Though SBR epidemics have not been a regular annual occurrence in the continental United States, *P. pachyrhizi* periodically causes severe and widespread disease outbreaks, causing intermittent issues for farmers throughout the United States (Zhang et al. 2012; Mundt et al. 2013). Variation in the severity of the United States SBR epidemics has been proposed to be due to varying environmental conditions and the amount of disease in Florida at the beginning of the soybean growing season (Christiano and Scherm 2007; Mundt et al. 2013). The main strategy for combating SBR in the United States is through timed fungicide applications. Resistance genes have been introgressed into elite soybean lines in the United States, and germplasm carrying SBR resistance genes is available (Boerma et al. 2011; Diers et al. 2014; King et al. 2016; Chicowski et al. 2024). However, commercial soybean cultivars resistant to SBR have not been deployed due to the challenge of selecting high-yield cultivars that carry native *Rpp* (resistance to *P. pachyrhizi*) genes (Childs et al. 2018). In countries such as Brazil, however, *P. pachyrhizi* has overcome genetically resistant soybean varieties within a few years of their *en masse* deployment throughout production regions (Hartman et al. 2005). These rapidly defeated soybean cultivars suggest that there may be either a high standing pool of *P. pachyrhizi* genetic diversity or that recombination is frequent enough in South American populations to overcome new host-resistant cultivars.

In March of 2001, severe SBR was observed in Paraguay and had spread throughout Paraguay and Brazil by May. SBR severity was lower in Paraguay due to drought, but spread to greater than 60% of the soybean acreage in Brazil resulting in substantial yield losses (Yorinori et al. 2005). Farmers in Brazil have attempted to manage *P. pachyrhizi* with a combination of early plantings, early maturing varieties, fungicides, and the deployment of new soybean rust resistance cultivars (Belay et al. 2023). In the United States, the history of SBR differs, and so does the approach to disease management. The amount of standing genetic variation in *P. pachyrhizi* populations and how it is partitioned across the landscape in the Southeastern United States is important to discern whether the *P. pachyrhizi* genetic diversity in the United States is comparable to, or different from, Brazil, where resistant soybean cultivars are quickly defeated. Mundt et al. (2013) argued and provided evidence that the severity of an SBR epidemic in the United States is determined by the severity of disease in Florida in late winter, after which disease spreads north over time ending in southern Canada in July or August. Consequently, it is important to understand the genetic diversity, population structure, phylogenetic relationships, and migration of *P. pachyrhizi* in the Southeastern United States. The data will provide insight into *P. pachyrhizi's* ability to locally adapt to environmental conditions in the United States (Paul et al. 2015). Additionally, they will help determine if *P. pachyrhizi* is diverging rapidly from earlier isolates identified closer to its introduction to the United States, and if there have been more recent introductions of *P. pachyrhizi* into the United States since 2004.

## Materials and methods

### *P. pachyrhizi* Isolates and Urediniospore Increase

**Fig. 1.**
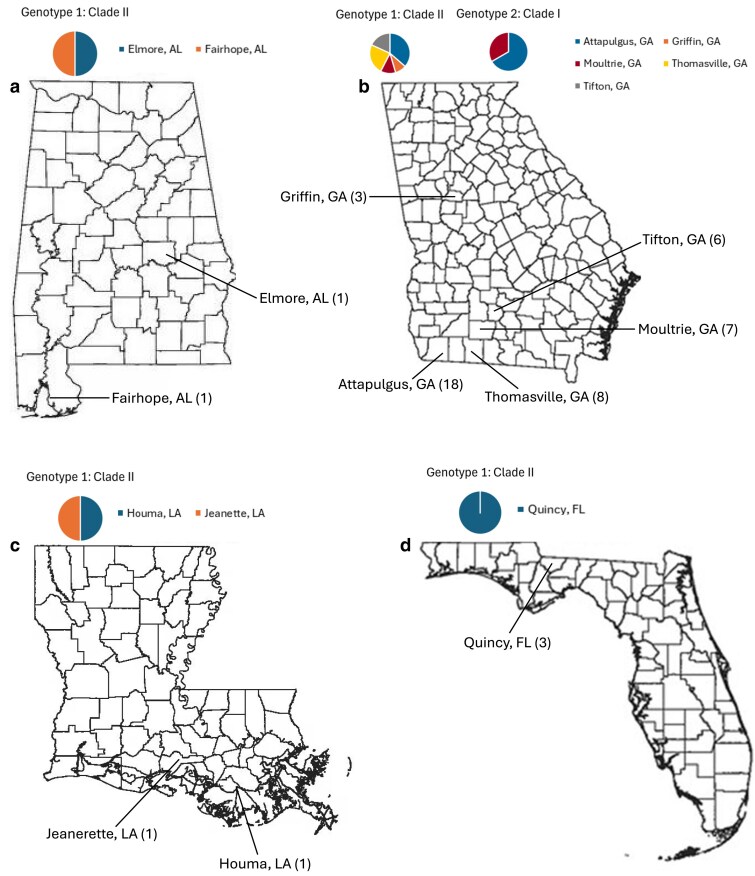
Geographic location of 49 *Phakopsora pachyrhizi* in four southeastern states. Colors correspond to the number of *P. pachyrhizi* isolates in Genotype 1: Clade II and/or Genotype 2: Clade I in a) Alabama, b) Georgia, c) Louisiana, and d) Florida. State maps were obtained from a public domain (https://www.teacherspayteachers.com/).

Forty-nine *P. pachyrhizi* isolates were collected from multiple localities during the 2008 to 2017 growing seasons including Georgia (forty-two isolates from five cities), Florida (three isolates from one city), Alabama (two isolates from two cities), and Louisiana (two isolates from two cities) (Table 1 and Fig. 1). These isolates were used in our study are a part of the southeastern soybean rust isolate collection (SESRIC). Isolates were collected predominately from multi-genotype sentinel soybean trial plots in south Georgia varying in traits to monitor soybean rust and other diseases. The Georgia isolates were bulk field isolates collected from multiple soybean leaves in a field exhibiting SBR symptoms. The Florida, Louisiana, and Alabama isolates were single uredial isolates maintained on a universally susceptible soybean variety (Williams 82) with at least three rounds of uredial reinoculations and were provided by David Walker (USDA-ARS Urbana, Illinois), Patricia Bollich (Louisiana State University), and Ed Sikora (Auburn University), respectively. To isolate single urediniospores for the Florida, Louisiana, and Alabama isolates, soybean seedlings were inoculated with a *P. pachyrhizi* field isolate by suspending each isolate in 0.04% Tween 20 and spraying the *P. pachyrhizi* urediniospore suspension on Williams 82. The newly inoculated soybean seedlings were placed in a growth chamber at 25 °C with high humidity and monitored daily for a well-isolated, non-erumpent pustule on a section of a leaf. When the pustule produced abundant urediniospores, single spores were collected with a small vacuum spore collector. Each of the single spore isolates was separately suspended in 0.04% Tween20 and used to inoculate a new seedling as described previously. Reinoculations were performed three times to maintain the isolate's genetic characteristics and prevent contamination from other rust strains or microorganisms.

**Table 1.** Forty-nine Phakopsora pachyrhizi isolates in the southeastern soybean rust isolate collection (SESRIC).

Isolate ID^a^	Isolate ID^a^	Isolate ID^a^
1. AL_Elmore_14	18. GA_Attapulgus_14.6	35. GA_Thomasville_13.1
2. AL_Fairhope_08	19. GA_Attapulgus_14.7	36. GA_Thomasville_14
3. FL_Quincy_09	20. GA_Attapulgus_14.8	37. GA_Thomasville_14.1
4. FL_Quincy_12	21. GA_Attapulgus_16	38. GA_Thomasville_14.2
5. FL_Quincy_15	22. GA_Attapulgus_16.1	39. GA_Thomasville_14.3
6. GA_Attapulgus_10	23. GA_Attapulgus_16.2	40. GA_Thomasville_16
7. GA_Attapulgus_13	24. GA_GRIF_13	41. GA_Thomasville_17
8. GA_Attapulgus_13.1	25. GA_GRIF_14	42. GA_Tifton_14
9. GA_Attapulgus_13.2	26. GA_GRIF_14.1	43. GA_Tifton_14.1
10. GA_Attapulgus_13.3	27. GA_Moultrie_13	44. GA_Tifton_14.2
11. GA_Attapulgus_13.4	28. GA_Moultrie_14	45. GA_Tifton_14.3
12. GA_Attapulgus_14	29. GA_Moultrie_14.1	46. GA_Tifton_14.4
13. GA_Attapulgus_14.1	30. GA_Moultrie_14.2	47. GA_Tifton_14.5
14. GA_Attapulgus_14.2	31. GA_Moultrie_14.3	48. LA_Houma_15
15. GA_Attapulgus_14.3	32. GA_Moultrie_14.4	49. LA_Jeanette_15
16. GA_Attapulgus_14.4	33. GA_Moultrie_14.5	
17. GA_Attapulgus_14.5	34. GA_Thomasville_13	

^a^First two letters in all isolate IDs correspond to the state the isolate was collected from (AL, Alabama; FL, Florida; GA, Georgia; and LA, Louisiana); Second designation corresponds to the city the isolate was collected in; Third designation corresponds to the year the isolate was collected (2008–2017 = _08-_17); Fifth designation corresponds to the isolate collection number (0.1 to 0.8).

Urediniospores with insufficient mass for DNA extractions were increased on detached leaves with Williams 82. First, three Williams 82 seeds were planted in 3.78-L pots that contained a soil mixture from SunGro Horticulture Inc. (Bellview, WA). Osmocote 15-9-12 was added on top of the potting mix. The pots were placed in a Conviron growth chamber set to 21 °C, 300 µmol sec^−1^ m^−2^ photosynthetically active radiation, and a day/night cycle of 16 h/8 h. Large trifoliate soybean leaves were detached from the soybean seedlings 21 d after planting. The detached leaves were washed briefly in distilled water and individually cut ∼1 cm from the base of the leaf to expose the vasculature. The detached leaves were placed in a 10 cm × 10 cm^2^ petri dish with sterilized cheesecloth at the bottom that was saturated with sterile distilled water. Cheesecloth was folded over the bottom 1/8 to 1/6 of the detached leaf to keep them from dehydration. The *P. pachyrhizi* urediniospore suspension was prepared separately for each of the isolates by suspending 5–10 × 10^4^ urediniospores mL^−1^ in ∼20 mL of 0.04% Tween20 solution and then vortexing to produce a homogenous spore suspension. Detached leaves were inoculated separately with each isolate by brushing the spore suspension onto the abaxial side of the leaves with a paintbrush or by pipetting the spore suspension onto the leaflet and distributing it over the leaf surface with a sterile gloved finger to ensure inoculation consistency between samples (JIRCAS 2019). Petri dishes containing the inoculated leaves were individually wrapped with parafilm and placed in a black plastic bag. After 24 h, petri dishes were removed from the black plastic bag and stacked on top of each other with half of each plate partially overlapping into a growth chamber with the same conditions described for the *P. pachyrhizi* urediniospore increase. *P. pachyrhizi* urediniospores were harvested from inoculated soybean leaves 14 to 21 d post-inoculation (dpi) in a biological safety cabinet with the laminar air flow off. Each isolate was harvested separately to prevent spore mixing and cross-contamination. Leaves with lesions were removed from the petri dish and gently tapped over a plastic weigh boat to dislodge urediniospores. A small paintbrush was used to remove the remaining urediniospores on the surface of the leaf. Spore-harvested leaves were placed back into the petri dish, sealed with parafilm, and returned to the growth chamber to encourage additional spore production. This process continued for each *P. pachyrhizi* isolate until the leaves ceased producing usable numbers of urediniospores. The harvested urediniospores were dried in a desiccant chamber for 24 h and stored in 1.5 mL Eppendorf tubes at −20 °C for short-term storage, then transferred to −80 °C for long-term storage.

### Urediniospore genomic DNA extraction

High molecular weight and high-quality DNA were extracted from the 49 SESRIC *P. pachyrhizi* isolates (2–20 mg of urediniospores) (Table 1) using the lysis method developed by Nagar and Schwessinger (2018). Urediniospores were placed in a precooled mortar with 1 g of autoclaved, acid-washed sand and liquid nitrogen, and ground with a pestle for 1–2 min. Urediniospores and sand were transferred to a 30 mL Beckman centrifuge tube with 5 mL of 2X CTAB extraction buffer, 140 µL of beta-mercaptoethanol, and 10 µL of proteinase K. The solution was heated in a 65 °C water bath for 30 min and then placed on a benchtop shaker at 70 rpm for 60 min. A phase separation followed with 5 mL of 24:1 chloroform:isoamyl alcohol, 30 min on the tabletop shaker at 70 rpm, and centrifugation in an Avanti Beckman centrifuge at 16,000 × *g* and 20 °C for 5 min. The upper aqueous phase was transferred to a new centrifuge tube, treated with RNase A (10 mg/mL), and placed on a benchtop shaker at 70 rpm for 75 min at room temperature. The phase separation step was repeated, and the upper aqueous phase was transferred to a new centrifuge tube. The DNA was then precipitated with one volume of isopropanol. The resulting solution was centrifuged at 13,300 × *g* for 10 min at 4 °C. The supernatant was discarded and the resulting pellet was washed with 70% ethanol by centrifugation at 13,300 × *g* for 5 min at 4 °C. The supernatant was discarded, and the ethanol wash of the pellet was repeated. After discarding the supernatant, the DNA pellet was allowed to air dry in a biological safety cabinet with laminar air flow at room temperature. The DNA pellet was resuspended in a 1× TE buffer. DNA quantity and quality were assessed for each isolate with a Nanodrop 1000 (Fischer Scientific), Qubit Fluorometer (Invitrogen), and gel electrophoresis. The DNA extracted from all *P. pachyrhizi* isolates was stored in a refrigerator at 4 °C until submitted for genotype-by-sequencing.

### Genotyping-by-sequencing (GBS)

GBS provided sequence data for downstream genetic diversity analyses for 49 high-quality *P. pachyrhizi* DNA samples for four Southeastern states (Alabama, Florida, Georgia, and Louisiana). A common restriction enzyme (*PstI*) and a rare cutter restriction enzyme (*MspI*) were used to generate uniform libraries that consisted of a forward, barcoded, adapter and a reverse Y-adapter on alternate ends of each fragment (Poland et al. 2012). This two-enzyme method was used to help manage the complexity of the large and repetitive *P. pachyrhizi* genome. Library preparations were conducted for sequencing on an Illumina NovaSeq 6000 platform with 150 bp paired-end reads. All library preparations and sequencing were performed at the Wisconsin Biotechnology Center at the University of Wisconsin.

### Sequence data analysis

**Fig. 2.**
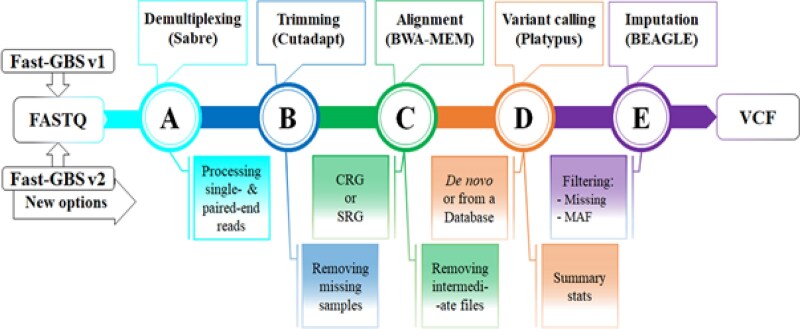
*Phakopsora pachyrhizi* isolate raw sequence data processing pipeline. A six-step Fast-GBS reference-based pipeline was used to process FastQ raw sequence data from the sequencing run to produce a VCF file for genetic diversity analysis: (1) Importing the raw sequence data, (2) a) Demultiplexing of pooled reads, (3) b) Filtering of low-quality reads and trimming of adapter sequences, (4) c) Alignment of the reads, (5) d) Variant calling and identification of polymorphisms in the aligned contigs, and (6) e) Imputation of missing data in the variant call format (VCF) file (Torkamaneh et al. 2017).

The annotated *P. pachyrhizi* draft genome generated by the Joint Genome Institute and Sérgio H. Brommonschenkel was used as a reference genome (*Phakopsora pachyrhizi*  MT2006 Standard Draft). The sequencing reads for each isolate were aligned to the *P. pachyrhizi* draft genome. Single-nucleotide polymorphisms (SNPs) were identified with the Fast-GBS v2 reference-based pipeline (Torkamaneh et al. 2017, 2019). This Fast-GBS referenced-based pipeline accomplished six major tasks: (1) importing the raw sequence data, (2) de-multiplexing of the pooled reads, (3) filtering of low-quality reads and trimming of adapter sequences, (4) alignment of the reads, (5) identification of polymorphisms in the aligned contigs, and (6) imputation of missing data in the variant call format (VCF) file (Fig. 2). Open-source tools within the pipeline were used to accomplish the following: (1) Sabre (demultiplexing) (Sabre 2019), (2) Cutadapt (read trimming and cleaning) (Martin 2011), (3) BWA (read mapping) (Li and Durbin 2010), (4) SAMtools (file conversion and indexing) (Li, 2011), (5) Platypus (post-processing of reads and variant calling) (Ewing and Green 1998; Rimmer et al. 2014), and (6) BEAGLE (missing data imputation and phasing of biallelic sites) (Browning and Browning 2007, 2016). Resulting variant calls were then used to generate consensus sequences that differed from the reference genome in genomic regions sampled by the GBS protocols using the R version 4.0.5 (R Core Team 2018) and vcfR 4.5 (Jombart and Ahmed 2011).

Adaptor contamination in each *P. pachyrhizi* isolate was identified using the run_fcsadaptor.sh script in the NCBI Foreign Contamination Screen (FCS) toolkit (v0.5.5) (Astashyn, et al. 2024). Adaptor sequences were cleaned from all *P. pachyrhizi* isolate genomes by splitting the scaffolds at the locations of contaminating sequences using fcs.py (options: clean genome). Cleaned genomes were verified to be adaptor free by applying a second round of run fcsadaptor.sh. All sequences in cleaned *P. pachyrhizi* isolate genomes were verified to be greater than 200 bp in length and not to contain N's at their beginning or end prior to submission to NCBI.

### Population genetic analyses

Filtered VCF files were analyzed in R version 4.0.5 (R Core Team 2018) with the packages vcfR 4.5 (Jombart and Ahmed 2011), poppr 2.0 (Kamvar et al. 2015), ape 5.8-1 (Knaus and Grünwald 2017), and adegenet 2.1.11 (Paradis and Schliep 2019). G_ST_ (The Coefficient of Genetic Differentiation) and heterozygosity values for the 49 *P. pachyrhizi* isolates at each geographic location were calculated in R using the genetic_diff function in the poppr package. G_ST_ was used as a measure of allelic frequencies across a population as a more appropriate alternative to F_ST_ (Fixation Index). F_ST_ measures genetic differentiation among and within populations. The standard of 0.0–0.05, 0.06–0.15, and 0.16–0.25 representing low, moderate, and high genetic differentiation among populations, respectively, was used for the biallelic model. A genotype rarefaction curve (in poppr 2.0) was calculated to determine the minimum number of SNPs needed to differentiate unique genotypes, identify how many multilocus genotypes (MLGs) were found among the isolate pools, and index how well the standing genetic diversity was represented in the isolate population by inspecting the slope of the rarefaction curve at 49 isolates (Grünwald 2003; Kamvar et al. 2015; Harrelson et al. 2021). We used Nei's genetic distance to construct a neighbor-joining distance-based tree to visualize the relationships among the *P. pachyrhizi* isolates as a dendrogram. G_ST_ values were calculated to compare divergence of subsets of the isolate groups inferred by the neighbor-joining tree. Discriminant analysis of principal components (DAPC) (Jombart et al. 2010), K-means hierarchical clustering in the program STRUCTURE (Hubisz et al. 2009), and Principal Coordinates Analysis (PCoA) with Nei's Genetic Distance and Bray-Curtis Dissimilarity Index were used to determine whether partitioning of the 49 isolates into groups was consistent among the methods.

## Results

### SNP calling, data filtering, and genetic diversity

**Fig. 3.**
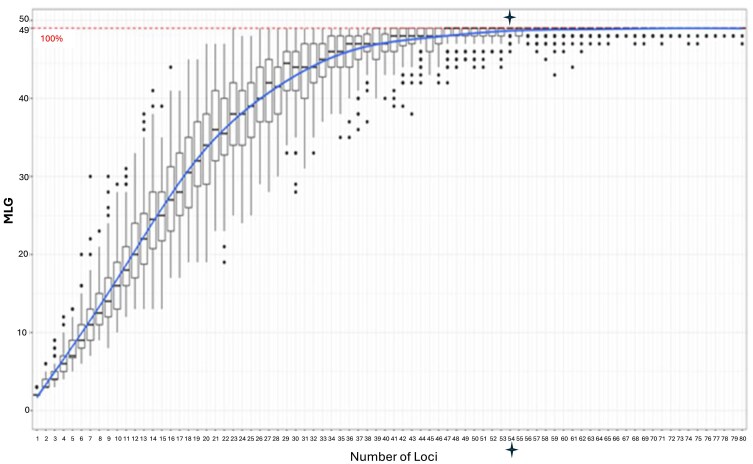
Genotype rarefaction curve for 49 *Phakopsora pachyrhizi* southeastern isolates constructed in poppr (version 2.9.1). Numbers on the *x*-axis indicate the number of loci. Numbers on the *y*-axis indicate the number of multilocus genotypes (MLG). The horozontal dotted line represents the maximum number of MLG identified in the 49 *P. pachyrhizi* isolates and extrapolates to the minimum number of SNPs on the *x*-axis (54 SNPs = blue stars) that would be necessary to differentiate each MLG in the collection of *P. pachyrhizi* isolates.

Fast-GBS was used in the current study to analyze the genetic diversity of 49 *P. pachyrhizi* isolates collected from four Southeastern states (Alabama, Florida, Georgia, and Louisiana) (Table 1) over a ten-year period. Raw sequence data contained 105,252 Mb of reads (Supplementary Table 1). Sequence reads had a mean quality score of 35.41, and 91.78% of reads had a quality score greater than 30. Consensus sequences for all *P. pachyrhizi* isolates were found to have adaptor contamination in some locations, which were inferred to arise from adaptor sequences present in the original reference genome and were therefore removed. The unfiltered VCF file contained 139,781 SNPs and, after filtering, 11,217 SNPs were retained and used for all analyses performed in this study (Fig. 2). Each isolate had a unique combination of SNPs, and the number of MLGs equaled the number of *P. pachyrhizi* isolates analyzed(Fig. 3). This analysis also revealed that all predicted MLGs could be differentiated with 54 SNPs (Fig. 3). The highest average allelic heterozygosity was observed for Georgia isolates collected from Attapulgus (0.263) and Moultrie (0.246) cities, while the lowest was observed for an Elmore, Alabama (0.219) isolate (Table 2). The average allelic heterozygosity and G_ST_ across all *P. pachyrhizi* isolates were 0.259 and 0.064, respectively.

**Table 2.** Geographical sources, average allelic heterozygosity (genetic diversity), and average G_ST_ value for 49 Phakopsora pachyrhizi southeastern isolates.

Field Location	Latitude/Longitude	General Location	Number of Isolates	Average Heterozygosity^a^
Attapulgus, GA	30.76′ N/84.48′ W	UGA Attapulgus Research and Education Center	18	**0**.**263**
Griffin, GA	33.26′ N/84.28′ W	UGA Griffin Campus	3	0.243
Moultrie, GA	31.13′ N/83.70′ W	SunBelt Ag Expo	7	**0**.**264**
Thomasville, GA	30.83′ N/83.97′ W	Commercial soybean field in SW Georgia	8	0.242
Tifton, GA	31.47′ N/83.53′ W	UGA Tifton Campus and Coastal Plain Experiment Station	6	0.244
Elmore, AL	32.35′ N/86.09′ W	East-central Alabama (north of Montgomery)	1	**0**.**219**
Fairhope, AL	30.32′ N/87.52′ W	Auburn University Gulf Coast Res. & Ext. Center (SW Alabama)	1	0.235
Quincy, FL	30.32′ N/84.35′ W	University of Florida North Florida Res. & Ed. Center (north-central FL)	3	0.252
Houma, LA	29.35′ N/90.42′ W	Terrebonne Parish in SE Louisiana (SW of New Orleans)	1	0.220
Jeanerette, LA All Locations All Locations	29.54′ N/91.40′ W All Locations All Locations	Iberia Parish in south-central Louisiana All Locations All Locations	1 49 49	0.229 0.259 0.064^b^ (Average G_ST_)
Attapulgus, GA; Moultrie, GA	30.76′ N/84.48′ W; 31.13′ N/83.70′ W	UGA Attapulgus Research and Education Center; SunBelt Ag Expo	9	0.02^c^ (Average G_ST_: Clade I Isolates)
All Locations	All Locations	All Locations	40	0.07^c^ (Average G_ST_; Clade II Isolates)

^a^Indicates the average heterozygosity distribution values for the *Phakopsora pachyrhizi* isolates at each location and across all geographic locations. Bold values indicate highest (Moultrie, GA) and lowest (Elmore, AL) average allelic heterozygosity observed for the 49 *P. pachyrhizi* isolates.

^b^Indicates the average G_ST_ value for the 49 *P. pachyrhizi* isolates across all geographic locations.

^c^Indicates the average G_ST_ value for the *P. pachyrhizi* isolates in clade I (9 isolates) and clade II (40 isolates) in the Neighbor-Joining rooted phylogenetic tree of 49 *P. pachyrhizi* isolates in Fig. 4.

### Neighbor-joining phylogenetic analysis of *P. pachyrhizi isolates*

**Fig. 4.**
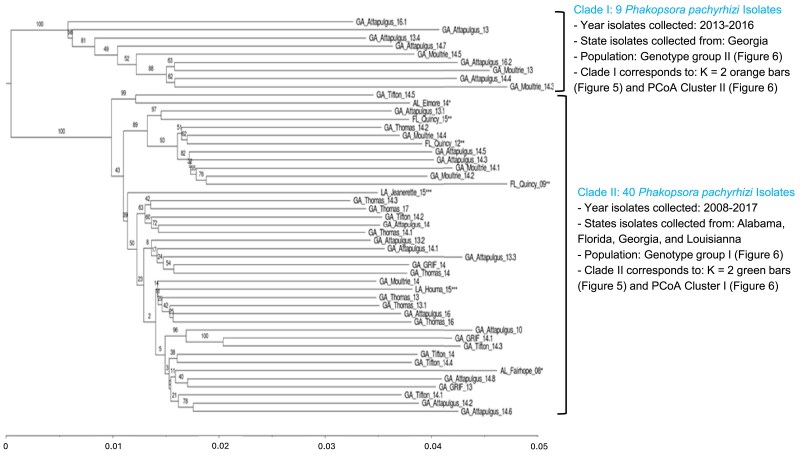
Neighbor-joining rooted phylogenetic tree of 49 *Phakopsora pachyrhizi* isolates collected from four southeastern states based on Nei's Genetic Distance. Isolate name corresponds to state (AL, Alabama; FL, Florida; GA, Georgia, and LA, Louisiana), collection city, collection year (2008–2017 = _8 to _17), and isolate collection number (0.1 to 0.8). An “* “indicates isolates from Alabama, “**” indicates isolates from Florida, and “***” indicates isolates from Louisiana. Numbers at the nodes are bootstrap values calculated from 1,000 resamplings.

Phylogenetic analysis using a neighbor-joining tree based on Nei's genetic distance calculations parsed the 49 *P. pachyrhizi* isolates in two well-supported clades with 100% bootstrap support (Fig. 4). Nine *P. pachyrhizi* isolates were clustered in clade I while the other 40 isolates clustered in clade II. Clade I contained isolates from Moultrie and Attapulgus, Georgia, and were collected from 2013 to 2016. Clade II contained all *P. pachyrhizi* isolates from Alabama (AL_Elmore_14, and AL_Fairhope_08), Florida (FL_Quincy_09**, FL_Quincy_12**, and FL_Quincy_15**), and Louisiana (LA, Jeanerette_15*** and LA_Houma_15***), and 33 Georgia isolates from Attapulgus, Griffin, Moultrie, Thomasville, and Tifton, that were collected from 2008 and 2017. All *P. pachyrhizi* Florida isolates and one of the Alabama isolates (AL_Elmore_14*) clustered with the eight Georgia isolates from Attapulgus, Moultrie, and Thomasville, in well-supported subclades in clade II with 99 and 89% bootstrap values. The two Louisiana isolates and the other Alabama isolate (AL_Fairhope_08) grouped with twenty-five Georgia isolates, although subclade bootstrap values were below the bootstrap cutoff of 75%. AL_Fairhope_08 and FL_Quincy_09 isolates were collected in 2008 and 2009, respectively, and were the oldest isolates in the collection. These two isolates clustered in clade II and were collected from 2008–2017. Isolates from south Georgia collected from 2008 to 2017 were also identified in clade II. *P. pachyrhizi* isolates in clades II were randomly subsampled to nine, the lowest number of isolates in clade I, and G_ST_ values calculated for each clade. The G_ST_ values for clade I and clade II were 0.02 and 0.07, respectively.

### Genetic subdivision of *P. pachyrhizi* isolates

**Fig. 5.**
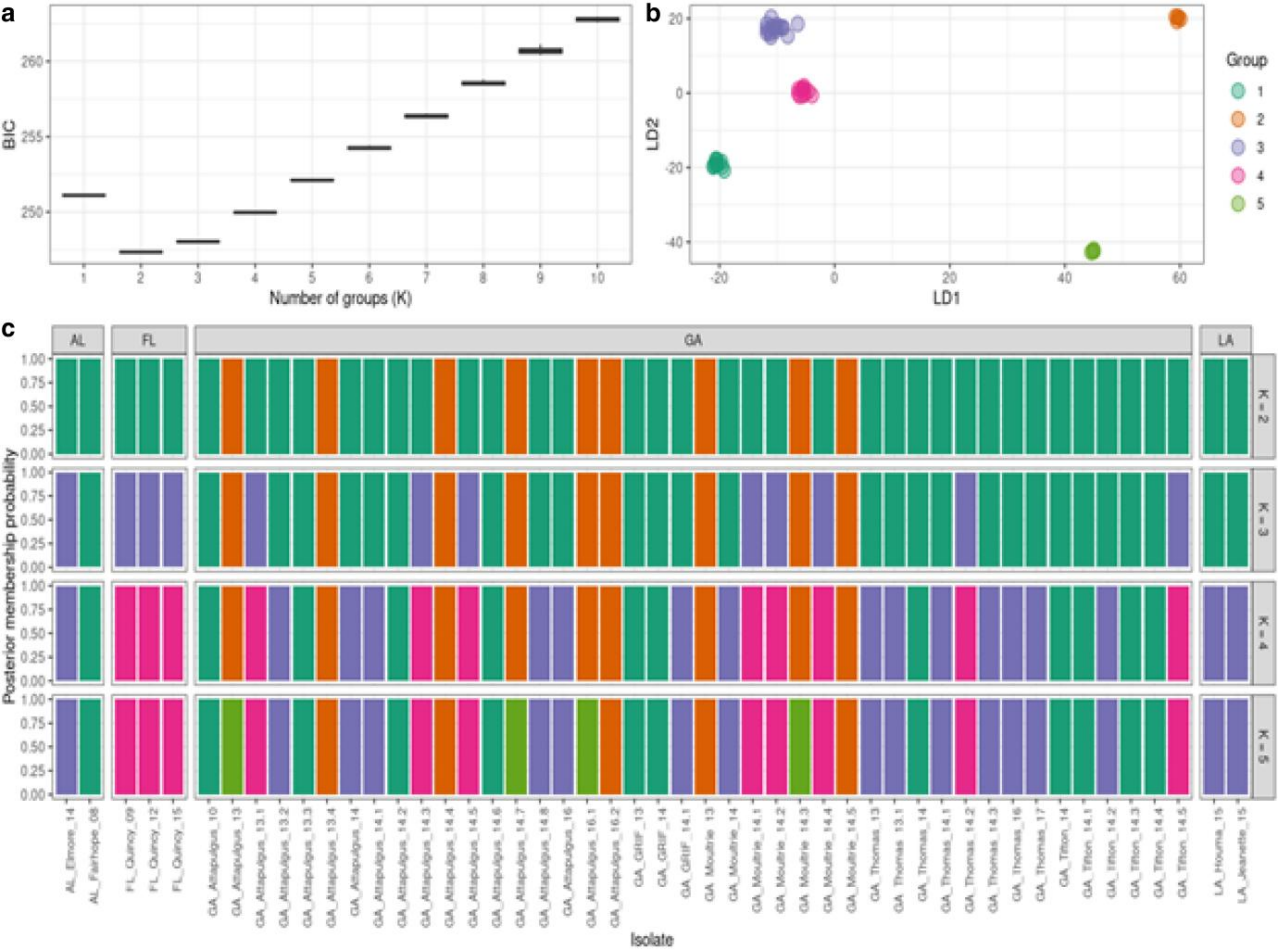
K-means hierarchical clustering and discriminant analysis of principal components (DAPC) analysis for 49 *Phakopsora pachyrhizi* southeastern isolates. a) K-means hierarchical clustering with K = 2 being the most favored model. The number of K = x groups are represented on the *x*-axis and the *y*-axis represents the Bayesian Information Criterion (BIC) scores. b) Individual isolate assignment probabilities to models evaluating K = 2–5 populations. The likelihood values are only positive on both axes for K = 2 groups. c) DAPC analysis of posterior membership probability of each sample if K = 2, 3, 4, and 5. Each row represents a different K value, with isolates divided into columns arranged by the isolate geographic location. All isolates were assigned 100% posterior membership probability to their respective assigned populations for K = 2–5 populations.

**Fig. 6.**
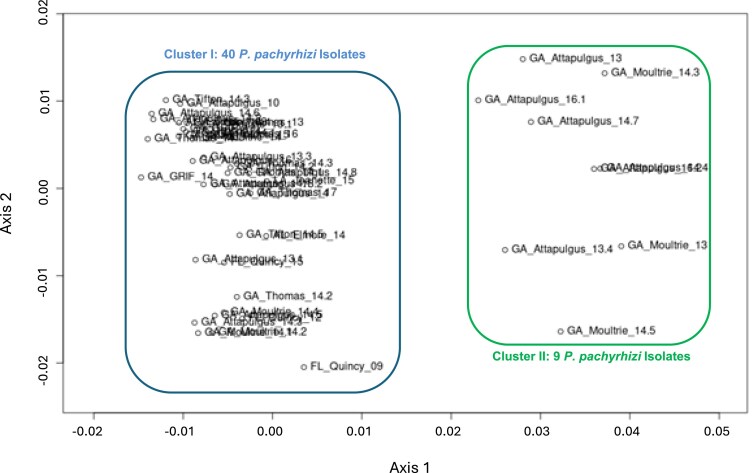
Principal coordinates analysis (PCoA) of 49 *Phakopsora pachyrhizi* southeastern isolates using Nei's Genetic Distance. The *x* and *y* axes represent ordination axes 1 and 2, and these axes describe 27 and 9% of the variation in the data, respectively. Isolates in Cluster 1 (Clade II) and Cluster II (Clade I) are the same isolates that grouped together in DAPC and the Neighbor-Joining tree and are in the same order (Fig. 4).

K-means hierarchcial clustering in the program STRUCTURE selected K = 2 populations as the model that best partitioned the 49 isolate genotype data (Fig. 5). Solutions for both K = 1 and K = 3 had much higher Bayesian Information Criterion (BIC) values compared with the model selecting K = 2 (Fig. 5). When K = 2, genotype assignments to one of two groups was relatively consistent (cluster I = green bars; cluster II = orange bars), whereas more variation with looser group assignments were made when the number of proposed K groups increased (Fig. 5). Additionally, the isolates partitioned into the two groups were the same isolates in clades I and II from the neighbor-joining tree (Fig. 4). In DAPC, when K = 3, 4, or 5, individuals within cluster I were separated into different clusters, but cluster II still remained as a well-defined group. Principal Coordinates Analysis (PCoA) yielded similar results regardless of whether we used Nei's genetic distance or Bray-Curtis dissimilarity index (Fig. 6, Supplementary Fig. 1). In both ordinations, the 49 *P. pachyrhizi* isolates appeared in two clusters of points, one on the left side of the ordination space and one on the right side with Axis 1 and Axis 2 explaining ∼ 36% of the variation in the data set (Fig. 6). The clusters in the PCoA ordinations were composed of the exact same isolates identified in the neighbor-joining tree, K-means clustering, and DAPC results.

## Discussion

We used GBS to assess the genetic diversity of 49 *P. pachyrhizi* isolates collected from Alabama, Florida, Georgia, and Louisiana over the 2008 to 2017 soybean growing seasons. GBS is a high-throughput reduced-representation genome sequencing in which genomic DNA is digested with restriction enzymes, and short fragments are amplified and sequenced *via* next-generation sequencing (Elshire et al. 2011; Andrews et al. 2016). This method uses restriction endonucleases that target a small portion of the genome, making it a low-cost, high-throughput, and one-step method for single-nucleotide polymorphism (SNP) discovery and genotyping of large numbers of samples (Elshire et al. 2011; Timilsina et al. 2022). The predominant reason for utilizing this sequencing method over others is that the *P. pachyrhizi* genome is large for a rust (∼1 Gb), and it has large sections of repetitive DNA that have made whole genome sequencing prohibitive in the past (Link et al. 2014). Though GBS can be used without a reference sequence, an annotated draft genome for *P. pachyrhizi* was available, making GBS sequence alignments more reliable for analyzing *P. pachyrhizi* genetic diversity, phylogenetic relationships, and distribution within the Southeastern United States.

The number of MLGs (49) equaled the number of *P. pachyrhizi* isolates which indicated that there were 49 different MLGs in the isolate pool and that the genetic diversity was well sampled as the slope at the accumulation curve terminus was slightly greater than zero. The average allelic heterozygosity (0.295) and G_ST_ (0.064) across all *P. pachyrhizi* isolates suggested moderate to low genetic diversity among the *P. pachyrhizi* isolates. The observed G_ST_ values are what would be expected with a largely clonal fungal pathogen that rarely undergoes sexual or parasexual recombination in nature. Pathogens that undergo sexual reproduction typically exhibit higher G_ST_ values, like *Septoria passerinii* that had a G_ST_ value of 0.238 and a highly structured population in the northern great plains (Lee and Neate 2007). Other fungal pathogens such as *Phytophtora infestans* exhibited population structure and diversity of sexually reproducing organisms, and G_ST_ values as high as 0.449 have been reported in wild *Solanum* spp. (Grünwald et al. 2001). Like *P. pachyrhizi,* predominately clonal organisms such as *Phaeoisariopsis griseola* also exhibited G_ST_ values as low as 0.004 (Mahuku et al. 2002). The lower G_ST_ values in our *P. pachyrhizi* population are in line with other predominately clonal populations and also indicate that more generalist fungal pathogen populations could be selected (Gillespie 1973). The link between lower G_ST_ values, implying low genetic differentiation between populations, and the selection for generalist fungal pathogen populations aligns with the concept that selection pressures can favor adaptability in variable environments. If different populations of a fungal pathogen like *P. pachyrhizi* encounter diverse host resistance or environmental conditions, the selection pressure may favor the emergence of generalist plant pathogens with the ability to infect a wider range of hosts. *P. pachyrhizi* produces only two types of spores: urediniospores, which are repeating asexual spores, and teliospores, which are spores that do not infect plants but function as survival spores in the life cycle (Hossain et al. 2024). Germination of teliospores to produce basidiospores is necessary for entering the sexual cycle but has not been observed in the wild. It is unclear if *P. pachyrhizi* can complete its lifecycle on a single host species or if it requires two or more host species to complete its lifecycle. There is evidence that *P. pachyrhizi* undergoes a parasexual cycle that involves somatic hyphal fusion and genetic recombination (Goellner et al. 2010; Li et al. 2019). This suggests that the *P. pachyrhizi* parasexual lifecycle, in the absence of sexual stages, is possibly a contributing factor to the moderate to low level genetic diversity observed for the 49 *P. pachyrhizi* isolates.

Because our number of isolates may be limiting with respect to fully characterizing the patterns and partitioning of SBR genetic diversity in the Southeast United States, we used multiple different analyses with different and sometimes unrelated algorithms to determine whether the patterns in the isolate GBS data were consistent. Additionally, we used multi-genotype soybean sentinel trial plots to simulate the complexity of a natural field environment to maximize the probability of sampling a wider array of *P. pachyrhizi* isolates from a single location. The majority of the *P. pachyrhizi* isolates were collected from five soybean sentinel trial plots in south Georgia that have multiple different soybean lines with varying traits including maturity group, resistance/susceptibility to different diseases, high-yielding cultivars, and high-oleic acid content. A diverse set of soybean genotypes, especially those with varying resistance traits, can create different selection pressures on the *P. pachyrhizi* population, allowing different *P. pachyrhizi* isolates to express their virulence on the most compatible host. Therefore, while sampling a limited number of *P. pachyrhizi* isolates, the differences we observed in the multi-genotype soybean sentinel trial plots may harbor a reservoir of *P. pachyrhizi* genotypes in a small area with greater genetic potential than would be found on a single soybean genotype planted across multiple fields or throughout a region. Neighbor-joining tree results suggested two distinct clades of isolates in the Southeastern United States, both of which appeared to be closely related to each other (low but detectable divergence). Separation of the 49 *P. pachyrhizi* isolates in two distinct clusters was supported by the DAPC analysis, K-means clustering, and the PCoA analysis. Despite the differing statistical approaches and diverse suite of algorithms, the same groups were indicated in each analysis and the isolate membership for each group was consistent with the neighbor-joining tree. These results indicate that the genetic divergence patterns within the *P. pachyrhizi* isolates were strong enough to be identified through different data analysis methods. We posited that if the same isolates were associated with the same groups in the suite of different statistical analyses, then the patterns of genetic differentiation and association are well-supported and likely representative of soybean rust genetic architecture in the Southeastern United States.

Georgia *P. pachyrhizi* isolates from the same locations and time periods clustered into different clades on the neighbor-joining tree indicating that they are genetically distinct. This suggests that at least two genetically different *P. pachyrhizi* populations or lineages (genotype I/clade I and genotype II/clade II) co-existed in the same geographic region during the same years (Fig. 1). This supports our hypotheses of either the potential for separate introduction events, one in 2004 and one following 2004 which remained geographically localized, or a simultaneous introduction event in 2004 of varied lineages where one group of lineages eventually became dominant over the other. In the latter scenario, the minority group of lineages may have persisted and evolved in soybean trial sentinel plots in south Georgia where many host lineages are confined to a small area potentially encouraging pathogen interaction and subsequent rust diversification. The DAPC analysis strongly suggested that there was no admixture among isolates in genotype groups I and II given any likely K number of groups. Adaptive and rapid geographically restricted partitioning of genetic diversity is known for other rust fungal pathogens where parasexuality, which we suspect based on our SBR isolate results, generated adaptive genetic diversity (Brasier and Kirk 2010; Lemaire et al. 2016; Asuke et al. 2021; Rahnama et al. 2023). Perhaps the most well-known example of rapid rust pathogen evolution occurred in a newly emerged strain (Ug99) of the wheat stem rust pathogen *Puccinia graminis f. sp. tritici* (*Pgt*). Ug99 was derived by somatic hybridization and nuclear exchange between dikaryons in parts of the world where the alternate host was absent or sparce and *Pgt* propagation was restricted to asexual propagation (Li, et al. 2019). The Ug99 strain persisted in the population and shared one haploid nucleus with the older African lineage of *Pgt*, and in this combination presented a substantial threat to the global wheat production through a significant increase in virulence and disease severity. This suggests that fungal pathogens that reproduce asexually can generate genetic diversity and facilitate the emergence of new lineages. The two *P. pachyrhizi* genotype groups have been apparently distinct from each other from at least 2013 to 2016, given that genotype group II (cluster II) contained isolates collected in those years (Fig. 6). When *P. pachyrhizi* was first found in the United States in 2004, it was hypothesized to be vectored to the United States on a hurricane (Bonde et al. 2006). The *P. pachyrhizi* isolate genotype represented in our study by clade II in the neighbor-joining tree is highly likely the genotype that arrived in the United States in 2004. Isolates in clade II are more diverse and were collected from 2008 to 2017. Isolates in clade I, which appear less diverse, likely arrived during or before 2013. Additionally, isolates in clade I were less prevalent in the Southeastern United States and may be due to low levels of inoculum maintained in the fields or under-sampling. Clade I isolates were not identified in Attapulgus and Moultrie, Georgia, in 2017 due to low levels of inoculum resulting from extremely hot and dry weather conditions in the two cities that year. Moreover, when *P. pachyrhizi* isolates in clade II were randomly subsampled to control for the sample size, clade I was still much less diverse than clade II. Together with the broader geographic distribution of clade II, the monophyly of both clades, and the presence of South Georgia isolates in each clade collectively suggest that our hypothesis of a single but simultaneous introduction of multiple genotypes followed by dominance is not well supported. Instead, our geographic and genetic analysis supports our hypothesis of a two-phase introduction: an initial introduction of clade II *P. pachyrhizi* isolates in 2004 that spread across southeast Georgia, followed by a second, later introduction of clade I *P. pachyrhizi* isolates.

Based on our results, it is difficult to explain how *P. pachyrhizi* isolates in clade I could have arisen in the United States at the same time as *P. pachyrhizi* isolates in clade II and not generated as much diversity or extensive spread, unless clade II *P. pachyrhizi* isolates had fitness advantages such as higher virulence, greater ability to adapt to local conditions, or a smaller, more resilient population size, that allowed them to outcompete clade I *P. pachyrhizi* isolates. Additionally, phylogenetic analyses of the *P. pachyrhizi* isolates demonstrated that distinct clades, or lineages, exist independently, a pattern that also supports a two-phase introduction of the pathogen over time and not a single introduction followed by mutations. These two genotype groups were also identified together only in southern Georgia. If additional isolates were collected from neighboring states, it is possible that isolates from both genotype groups would be identified in those states. While southern Georgia is not the only overwintering source of inoculum, its position and favorable conditions mean it is a significant contributor of inoculum for the wider North American SBR epidemics, serving as one part of a larger, regional Southeastern United States inoculum source. Considering both the results from our study and the published literature on rust genetics, it appears that the Southeastern United States can potentially harbor new *P. pachyrhizi* genotypes if they are introduced and possibly generate new pathogenic races which may disperse from Florida and Georgia.

Understanding the spatial distribution of SBR isolates is important for tracking the spread and genetic diversity of the *P. pachyrhizi*. For example, Attapulgus Georgia isolates were collected from a field located in Southwest Georgia in the vicinity of the Georgia-Florida border (30.76′ N/84.48′ W). The Moultrie Georgia isolates were collected from a field about 50 miles northeast of the Attapulgus Georgia field closer to the center of Southwest Georgia (31.13′ N/83.70′ W) (Fig. 1). The distribution of SBR isolates in Southwest Georgia highlights the pathogen's movement within a connected agricultural zone. SBR isolates collected from fields in Attapulgus and Moultrie, located about 50 miles apart, confirm that SBR can spread locally. This is particularly significant in a region like Southwest Georgia, which has a favorable climate for fungal diseases and experiences intensive agriculture. As a regional hub of intensive agriculture, the landscape consisting of interconnected fields and common crop rotations allows for a contiguous environment where diseases like SBR can easily move from one field to another. The movement of SBR between Attapulgus and Moultrie underscores why monitoring networks and sentinel plots are essential for tracking the disease and providing early warnings to farmers. The data collected from sentinel plots provides valuable information on the pathogen's biology and spread.

Mundt et al. (2013) evaluated the expansion of the SBR epidemic during a seven-year period and demonstrated that the weather and the severity of the disease in the Southeastern United States determined the variation in the severity of the United States soybean rust epidemics. Because of this relationship, it is crucial to understand *P. pachyrhizi's* genetic diversity, adaptive changes, the partitioning of standing genetic diversity, and the introduction/generation of new genetic diversity in the Southeastern United States. SBR outbreaks in the Southeastern United States, while capable of long-distance dispersal through winds, do not yet produce predictable annual epidemics, but occur more frequently as locally intense outbreaks distributed across regions of the United States, which can be partially subdued by fungicide application. Although there is obvious potential for continental-scale SBR epidemics in the United States and severe occasional epidemics have occurred in the past (Mundt et al. 2013), the scenario in the United States is relatively mild compared with SBR epidemics in Brazil. Brazil uses a combination of genetically resistant soybean cultivars and large fungicide inputs to control *P. pachyrhizi* (Godoy et al. 2016). The genetically resistant soybean cultivars used in Brazil do not mitigate *P. pachyrhizi* outbreaks in the field on their own and must be combined with other management strategies, indicating that selection pressure and environmental conditions are two factors that influence *P. pachyrhizi* to propagate unrestricted across their production region. The genetic diversity and pathogenicity of twenty-three *P. pachyrhizi* mono-uredinial isolates collected in Brazil demonstrated high genetic diversity when assessing polymorphisms among AFLP markers locally and Internal Transcribed Spacer (ITS) sequences globally (Darben et al. 2020). The ITS region can be useful for identifying species within a closely related group but depending on the organism studied, might not be ideal for genetic diversity analysis due to its ability to rapidly evolve and maintain a high degree of sequence variation even among closely related individuals. This makes it challenging to accurately assess genetic relationships with the isolates from Brazil with the ITS region and AFLP markers (Bradshaw et al. 2023). We do not know how the partitioning of genetic diversity in the southeast United States compares with Brazil as only the ITS regions of the isolates from Brazil are available for comparison to our isolate genomic sequences from the United States and there are multiple ITS copies. If the patterns of *P. pachyrhizi* genetic diversity are similar, it suggests that SBR could become an annual and severe issue for the United States soybean producers.

In summary, moderate to low genetic diversity was observed among the *P. pachyrhizi* isolates, which is expected with a largely clonal fungal pathogen that rarely undergoes sexual or parasexual recombination in nature. The *P. pachyrhizi* population in the Southeastern United States is characterized by two distinct lineages representing two related genotype groups, suggesting separate introduction events with an initial arrival of a distinct lineage in 2004, likely Clade II, followed by a later introduction of the Clade I lineage. The population structure of *P. pachyrhizi* isolates in the Southeastern United States was due to geographic location not the year of isolate collection. SBR disease severity in the United States is not as severe as Brazil, however, there is a need to characterize and compare the patterns of genetic diversity within the United States and Brazil. Genetic diversity and virulence in the *P. pachyrhizi* population are required for pathogen adaptation, flexibility, and the ability to evolve in response to environmental changes (Hossain et al. 2024). It is not clear how *P. pachyrhizi* population diversity and structure contribute to *P. pachyrhizi* fitness or disease phenotypes. This presents an opportunity to evaluate the relationship between *P. pachyrhizi* pathogen diversity and virulence which will provide a better understanding of pathogen migration, emergence of new *P. pachyrhizi* strains, and pathogen population dynamics that are necessary to effectively manage SBR in the United States.

## Supplementary Material

jkaf267_Supplementary_Data

## Data Availability

The statistics of the Fast-GBS data underlying this article and the accession numbers are available in the supplemental material (Supplementary Table 1). All sequences have been deposited at the NCBI database (Accession numbers: JBPJSS000000000–JBPJUO000000000) and FigShare (https://doi.org/10.25387/g3.30521555). Supplemental material available at G3 online.
