# Supplementary material for: Population structure and genetic diversity of Phakopsora pachyrhizi in the Southeastern United States
Source: G3 (Bethesda). 2025 Dec 8;16(1):jkaf267. doi: 10.1093/g3journal/jkaf267 (PMC12774594; doi:10.1093/g3journal/jkaf267)
Supplement: jkaf267_Supplementary_Data [file jkaf267_supplementary_data.docx]

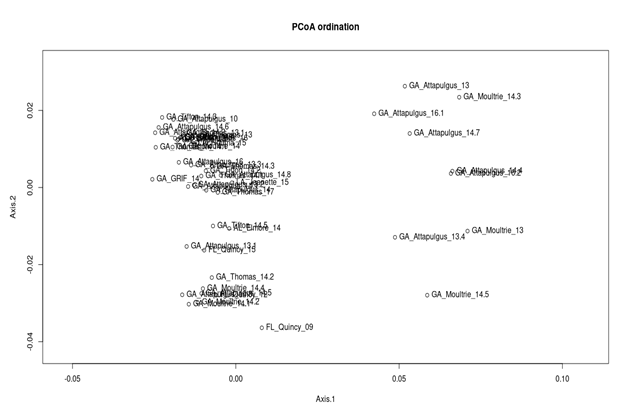


Cluster I: 40 *P. pachyrhizi* Isolates

Cluster II: 9 *P. pachyrhizi* Isolates

**Supplementary Figure S1.** Principal coordinates analysis (PCoA) of 49 *Phakopsora pachyrhizi* southeastern isolates using Bray-Curtis dissimilarity matrix as an alternative to Nei’s Genetic Distance. The x and y axis represent ordination axes 1 and 2 that describe 27% and 9% of the variation in the isolates, respectively. Isolates in Cluster 1 (Clade II) and Cluster II (Clade I) are the same isolates that grouped together in DAPC and the Neighbor-Joining tree and are in the same order (Figure 4).

| **Supplementary Table S1**. Statistics of sequencing data from 49 *Phakopsora pachyrhizi* southeastern isolates. | | | | | | |
| --- | --- | --- | --- | --- | --- | --- |
| **Accession Number** | **Isolate Id** | **Effective**  **Rate (%)** | **Error**  **Rate (%)** | **GC**  **Content (%)** | **Q20 (%)** | **Q30 (%)** |
| JBPJSS000000000 | AL_Elmore_14 | 100 | 0.03 | 44.54 | 97.68 | 91.95 |
| JBPJST000000000 | AL_Fairhope_08 | 99.97 | 0.03 | 47.99 | 98.9 | 92.33 |
| JBPJSU000000000 | FL_Quincy_09 | 100 | 0.03 | 45.26 | 96.78 | 93.02 |
| JBPJSV000000000 | FL_Quincy_12 | 100 | 0.03 | 42.68 | 98.28 | 87.54 |
| JBPJSW000000000 | FL_Quincy_15 | 100 | 0.03 | 42.78 | 97.55 | 93.23 |
| JBPJSX000000000 | GA_Attapulgus_10 | 100 | 0.03 | 42.77 | 97.68 | 94.52 |
| JBPJSZ000000000 | GA_Attapulgus_13 | 100 | 0.03 | 41.6 | 97.99 | 89.89 |
| JBPJTA000000000 | GA_Attapulgus_13.1 | 100 | 0.03 | 41.82 | 98.92 | 93 |
| JBPJTB000000000 | GA_Attapulgus_13.2 | 99.97 | 0.03 | 41.25 | 97.34 | 93.1 |
| JBPJTC000000000 | GA_Attapulgus_13.3 | 100 | 0.03 | 42.22 | 97.45 | 93.25 |
| JBPJSY000000000 | GA_Attapulgus_13.4 | 100 | 0.03 | 41.65 | 97 | 91.02 |
| JBPJTE000000000 | GA_Attapulgus_14 | 100 | 0.03 | 41.76 | 98.1 | 88.01 |
| JBPJTF000000000 | GA_Attapulgus_14.1 | 99.98 | 0.03 | 44.25 | 96.62 | 90.89 |
| JBPJTG000000000 | GA_Attapulgus_14.2 | 100 | 0.03 | 41.78 | 97.22 | 93.65 |
| JBPJTH000000000 | GA_Attapulgus_14.3 | 100 | 0.03 | 44.83 | 96.65 | 91.81 |
| JBPJTI000000000 | GA_Attapulgus_14.4 | 100 | 0.03 | 44 | 98.41 | 92.02 |
| JBPJTJ000000000 | GA_Attapulgus_14.5 | 100 | 0.03 | 42.22 | 98 | 90.62 |
| JBPJTK000000000 | GA_Attapulgus_14.6 | 100 | 0.03 | 43.25 | 98.54 | 93.03 |
| JBPJTL000000000 | GA_Attapulgus_14.7 | 100 | 0.03 | 44.44 | 98.16 | 92.89 |
| JBPJTD000000000 | GA_Attapulgus_14.8 | 100 | 0.03 | 45.89 | 97.87 | 89.09 |
| JBPJTN000000000 | GA_Attapulgus_16 | 99.97 | 0.03 | 44.1 | 97.65 | 89.97 |
| JBPJTO000000000 | GA_Attapulgus_16.1 | 100 | 0.04 | 43.63 | 96.01 | 90.09 |
| JBPJTM000000000 | GA_Attapulgus_16.2 | 100 | 0.03 | 45.26 | 98.81 | 92.68 |
| JBPJTP000000000 | GA_GRIF_13 | 99.97 | 0.03 | 44 | 95.66 | 90.45 |
| JBPJTQ000000000 | GA_GRIF_14 | 100 | 0.03 | 41.25 | 97.25 | 90.32 |
| JBPJTR000000000 | GA_GRIF_14.1 | 99.97 | 0.04 | 45.31 | 98.56 | 92 |
| JBPJTS000000000 | GA_Moultrie_13 | 100 | 0.03 | 41.55 | 97.64 | 90.56 |
| JBPJTU000000000 | GA_Moultrie_14 | 100 | 0.03 | 44.91 | 95.78 | 89.45 |
| JBPJTV000000000 | GA_Moultrie_14.1 | 100 | 0.03 | 44.45 | 96.96 | 92 |
| JBPJTW000000000 | GA_Moultrie_14.2 | 100 | 0.03 | 43.63 | 97.45 | 93.33 |
| JBPJTX000000000 | GA_Moultrie_14.3 | 99.96 | 0.03 | 41.25 | 94.85 | 87.69 |
| JBPJTY000000000 | GA_Moultrie_14.4 | 100 | 0.03 | 43.33 | 97.98 | 93.33 |
| JBPJTT000000000 | GA_Moultrie_14.5 | 100 | 0.03 | 41.01 | 98.89 | 92.54 |
| JBPJUA000000000 | GA_Thomasville_13 | 100 | 0.03 | 43.6 | 98.82 | 90.89 |
| JBPJTZ000000000 | GA_Thomasville_13.1 | 100 | 0.03 | 45.11 | 97.89 | 90.11 |
| JBPJUC000000000 | GA_Thomasville_14 | 100 | 0.03 | 44.89 | 97.01 | 92.45 |
| JBPJUD000000000 | GA_Thomasville_14.1 | 100 | 0.03 | 44.69 | 98.89 | 91.73 |
| JBPJUE000000000 | GA_Thomasville_14.2 | 100 | 0.03 | 44.52 | 98.81 | 89 |
| JBPJUB000000000 | GA_Thomasville_14.3 | 100 | 0.03 | 41.56 | 97.85 | 91.2 |
| JBPJUF000000000 | GA_Thomasville_16 | 100 | 0.03 | 43.58 | 97.54 | 89.1 |
| JBPJUG000000000 | GA_Thomasville_17 | 100 | 0.03 | 44.2 | 97.2 | 90.5 |
| JBPJUI000000000 | GA_Tifton_14 | 100 | 0.03 | 42.42 | 98.5 | 90.61 |
| JBPJUJ000000000 | GA_Tifton_14.1 | 100 | 0.03 | 43.9 | 97.45 | 89.98 |
| JBPJUK000000000 | GA_Tifton_14.2 | 100 | 0.03 | 44.61 | 98.81 | 92 |
| JBPJUL000000000 | GA_Tifton_14.3 | 100 | 0.03 | 41.25 | 98.02 | 92.36 |
| JBPJUM000000000 | GA_Tifton_14.4 | 100 | 0.03 | 49.25 | 98.24 | 92.55 |
| JBPJUH000000000 | GA_Tifton_14.5 | 99.98 | 0.03 | 44.25 | 98.25 | 92 |
| JBPJUN000000000 | LA_Houma_15 | 100 | 0.03 | 45.25 | 97.54 | 91.05 |
| JBPJUO000000000 | LA_Jeanette_15 | 100 | 0.03 | 45.55 | 97.77 | 90.19 |
